# *Pseudomonas aeruginosa* Cytotoxicity Is Attenuated at High Cell Density and Associated with the Accumulation of Phenylacetic Acid

**DOI:** 10.1371/journal.pone.0060187

**Published:** 2013-03-29

**Authors:** Jianhe Wang, Yihu Dong, Tielin Zhou, Xiaoling Liu, Yinyue Deng, Chao Wang, Jasmine Lee, Lian-Hui Zhang

**Affiliations:** 1 Institute of Molecular and Cell Biology, Singapore; 2 Department of Biological Sciences, National University of Singapore, Singapore; East Carolina University School of Medicine, United States of America

## Abstract

**Background:**

*P. aeruginosa* is known to cause acute cytotoxicity against various human and animal cells and tissues.

**Methodology/Findings:**

Intriguingly, however, in this study we noticed that while a low cell density inoculum of *P. aeruginosa* caused severe cytotoxicity against human lung tissue cell line A549, increasing the cell density of bacterial inoculum led to decreased cytotoxicity. Addition of the supernatants from high density bacterial culture to low cell density inoculum protected the human cells from bacterial cytotoxic damage, suggesting that *P. aeruginosa* may produce and accumulate an inhibitory molecule(s) counteracting its pathogenic infection. The inhibitor was purified from the stationary-phase culture supernatants of *P. aeruginosa* strain PAO1 using bioassay-guided high performance liquid chromatography (HPLC), and characterized to be phenylacetic acid (PAA) by mass spectrometry and nuclear magnetic resonance spectroscopy. Microarray analysis revealed that treatment of *P. aeruginosa* with PAA down-regulated the transcriptional expression of Type III secretion system (T3SS) genes and related regulatory genes including *rsmA* and *vfr*, which were confirmed by transcriptional and translational analysis.

**Conclusions:**

Identification of bacterial metabolite PAA as a T3SS-specific inhibitor explains this intriguing inverse cell-density-dependent-cytotoxicity phenomenon as T3SS is known to be a key virulence factor associated with cytotoxicity and acute infection. The findings may provide useful clues for design and development of new strategies to combat this formidable bacterial pathogen.

## Introduction

*Pseudomonas aeruginosa* is an important opportunistic human bacterial pathogen that can cause severe infections in cystic fibrosis patients and immuno-compromised individuals [1], [2]. The pathogen has evolved and utilizes various virulence mechanisms to gain competitive advantages over its host in acute and chronic infections. Among them, type III secretion system (T3SS) is a key virulence determinant that plays a critical role in establishing acute infection. This protein secretion and delivery system acts by injecting effector proteins into host cells, and with which to modulate the host cellular activities in favor of infection [3], [4], [5]. T3SS is known to play various roles in host-pathogen interaction, including generation of pores in host cells and promoting bacterial internalization [6], [7], [8], induction of macrophage apoptosis [9], and inhibition of phagocytosis by changing the structure of macrophage actin skeleton [10].

*P. aeruginosa* is known to contain about 43 T3SS genes but the number may vary slightly in a strain-dependent manner [11]. The transcriptional expression of these T3SS genes is coordinated by its master regulator ExsA, which activates the T3SS expression by binding to the conserved motif of T3SS gene promoters [11], [12]. The expression and function of ExsA are further modulated by several upstream regulators and signaling mechanisms, including cyclic AMP (cAMP) and cAMP-dependent global regulator Vfr [13], RetS/LadS/GacAS two-component regulatory systems [14], [15], [16], [17], host signals spermidine and spermine [18], and the small RNA binding protein RsmA [19]. These signaling and regulatory mechanisms function by either activating or repressing the T3SS expression of *P. aeruginosa* in response to intracellular and extracellular environmental cues.

Our previous study showed that maximum T3SS expression occurs at the early growth stage, which is then declined rapidly at the stationary growth phase [18]. However, little is known about the mechanisms involved in the arrest of the positive activation loop of the bacterial T3SS. Recently, it was reported that addition of the stationary-phase culture supernatants to exponential phase growing *P. aeruginosa* can inhibit T3SS expression [20]. Subsequent transposon mutagenesis analysis showed that null mutation of tryptophan synthase TrpA abolished the T3SS-inhibitory activity [20]. As tryptophan is the precursor of indole-3-acetic acid (IAA), IAA and its analogue 1-naphthalacetic acid (NAA) were then tested and found to be able to inhibit the T3SS expression of *P. aeruginosa* when added at a final concentration of 1 mM. However, IAA was not detectable in the stationary-phase culture supernatants of *P. aeruginosa* [20], indicating that the observed putative T3SS-inhibitory molecule may not be IAA.

In this study, we noticed that the virulence of PAO1 was attenuated when a high-cell-density inoculum was used to challenge the A549 cell line derived from human lung tissues. Subsequent studies showed that the attenuated virulence was due to the presence of an inhibitory molecule(s) in the supernatants of high-cell-density bacterial cultures. The inhibitor was purified by using high performance chromatography and its chemical structure was determined by using mass spectrometry and nuclear magnetic resonance (NMR) spectroscopy. Microarray and genetic analyses were further conducted to investigate the inhibitory mechanism of the identified inhibitor phenylacetic acid (PAA) on bacterial virulence. Our findings present a new insight to the puzzle of high-cell-density-modulated virulence attenuation in *P. aeruginosa* and the regulatory mechanisms of T3SS which is associated with bacterial acute infection.

## Materials and Methods

### Bacterial Strains and Culture Conditions

Bacterial strains and plasmids used in this study are listed in Table S1 (in File S1). Bacteria were routinely grown at 37°C in Luria-Bertani broth (LB) unless otherwise indicated. For induction of T3SS expression, LB medium was supplemented with the chelating reagent nitrilotiracetic acid (NTA) at a final concentration of 7.5 mM. Antibiotics were added at the following concentrations when required: kanamycin, 100 mg ml^−1^; rifampicin, 50 mg ml^−1^, tetracycline, 10 mg ml^−1^. 5-Bromo-4-chloro-3-indolyl β-D-glucopyranoside (X-gluc) was included in medium at a final concentration of 60 mg ml^−1^ for detection of β-glucuronidase (GUS) activity.

### Cytotoxicity Assay

To determine the cytotoxicity of PAO1, A549 cells were seeded in 96-well tissue culture plates containing 100 µl of Dulbecco’s Modified Eagle Medium (DMEM) and allowed to grow at 37°C for 16 to 18 h to obtain 80 to 90% monolayer confluency (about 1.0×10^4^ cells/well). Culture supernatants were removed, the monolayer was washed once with PBS buffer. For inoculation, the fresh bacterial cells were resuspended and diluted in DMEM or LB medium or culture supernatants as indicated to a concentration about 1×10^7^ CFU per ml or otherwise indicated. Thereafter, 100 µl of the bacterial dilution were applied to the A549 cell monolayers at a multiplicity of infection (MOI) of 50. After infection for 4 h at 37°C, A549 cell viability was determined by WST-1 assay, which quantifies mitochondrial metabolic activity, following the manufacturer’s instructions (Roche).

### Preparation of Bacterial Supernatants and Purification of Inhibitory Compound

*P. aeruginosa* strain PAO1 was grown overnight in LB as starter culture. Bacterial cells were washed twice with fresh LB medium and then inoculated at 1∶200 ratio in fresh LB and grown at 37°C with agitation at 260 rpm. For preparation of bacterial supernatants for assay of inhibitory activity, bacteria were removed at different cell densities as indicated by centrifugation at 8,000 g for 10 min at room temperature. Supernatants were collected after second round centrifugation at 15,000 g for 20 min at 4°C and sterilized by using 0.2 µm membrane filters. For purification, strain PAO1 was cultured under the same conditions till OD_600_ = 1.5. Two liters of bacterial supernatants were acidified to pH 3.5 by addition of HCl and extracted with an equal volume of ethyl acetate. The organic phase was evaporated and the residues were dissolved in 2 ml of methanol.

The crude extracts were separated by column chromatography using a Sephadex LH20 column, eluted with methanol. The active fractions were combined and separated by high performance chromatography (HPLC) on a C_18_ reverse-phase column (4.6 × 250 mm, Waters), eluted with acetonitrile–water gradient (80∶20 v/v) at a flow rate of 1 ml min^−1^. The absorbance spectra were recorded by Waters 2487 Dual λ Absorbance Detector and detected peaks were collected and assayed for the activity to inhibit PAO1 cytotoxisity on A549 cell line as described in the next section.

### Chemical Structure Analysis

NMR spectra were obtained on a Bruker Avance instrument (_1_H NMR at 500 MHz; _13_C NMR at 125 MHz), with chemical shifts being referenced to the solvent peak. The product was further confirmed by HRMS, electrospray ionization with a negative polarity (MicrOTOF-QII). High resolution mass spectra (HRMS, electron impact) were obtained at 20–40 eV.

### Quantitative β-galactosidase Assay

Overnight bacterial cultures were diluted 1∶200 to fresh LB medium supplemented with NTA as indicated. The growth was continued with shaking at 37°C for 4 h to allow OD_600_ reaching about 1.2. β-Galactosidase activity was measured as previously described [21]. The experiment was repeated for at least three times and the data shown are the means of three replicates and given as Miller units (MU).

### RNA Extraction and Microarray Analysis

Total RNA samples were isolated from fresh bacterial cultures using the RNeasy mini kit according to the manufacturer’s instructions (Qiagen), and digested with DNase I (Promega) to remove contaminating genomic DNA. The enzyme was then removed by RNeasy column purification. The quantity and purity of RNA were determined by agarose gel electrophoresis and UV spectrometry. cDNA was synthesized from total RNA samples by using random primers (Invitrogen). SuperScript II (Invitrogen) and biotin-ddUTP were used to label the products according to the protocol from Affymetrix. Target hybridization, washing and staining were performed following the manufacturer’s instructions. GeneChip arrays were scanned with an Affymetrix probe array scanner. The microarray analysis for each bacterial strain was repeated for three times and the data were analyzed using a statistics software MAS-5.0 from Affymetrix. The microarray data were provided in the Supplementary information and deposited in the NCBI GEO data base under accession No.: GSE43641.

### Real Time RT-PCR

RNA was purified using the Invitrogen PureLink kit, DNase-treated, quantified, and cDNA synthesized (GoScript Reverse Transcription system, Promega). Real time RT-PCR was performed using SYBR Green PCR Master Mix (Invitrogen) on LightCycler 4.0. The PCR primers used in this study were designed based on the genome sequence of *P. aeruginosa* strain PAO1 (http://www.pseudomonas.com) [22], which are listed in Table S2 (in File S1).

### Protein Isolation and Western Blotting Analysis

Overnight bacterial cultures were inoculated at a 1∶200 ratio to fresh LB medium supplemented with NTA or PAA as indicated. After incubation at 37°C for 4 h, the bacterial cultures were chilled on ice for 10 minutes. For each bacterial culture, 10 ml were taken and centrifuged. The supernatants and the bacterial pellets were used for preparation of extracellular and total cellular proteins, respectively. The supernatants were filtered with 0.2 µm syringe filter and precipitated with trichloroacetic acid (TCA) at a final concentration of 10%. The precipitates were pelleted by centrifugation, washed twice with acetone, dried, and re-suspended in SDS sampling buffer. For isolation of total cellular proteins, the bacterial pellets were resuspended in PBS buffer and the cells were broken by sonification. After centrifugation, the supernatants which contain total cellular proteins were kept for further analysis. The protein samples were denatured by boiling for 5 minutes and separated by 10% SDS-PAGE. Western blot analysis was performed following the standard protocol.

## Results

### Increasing the Cell Density of *P. aeruginosa* Inoculum Results in Attenuated Virulence

**Figure 1:**
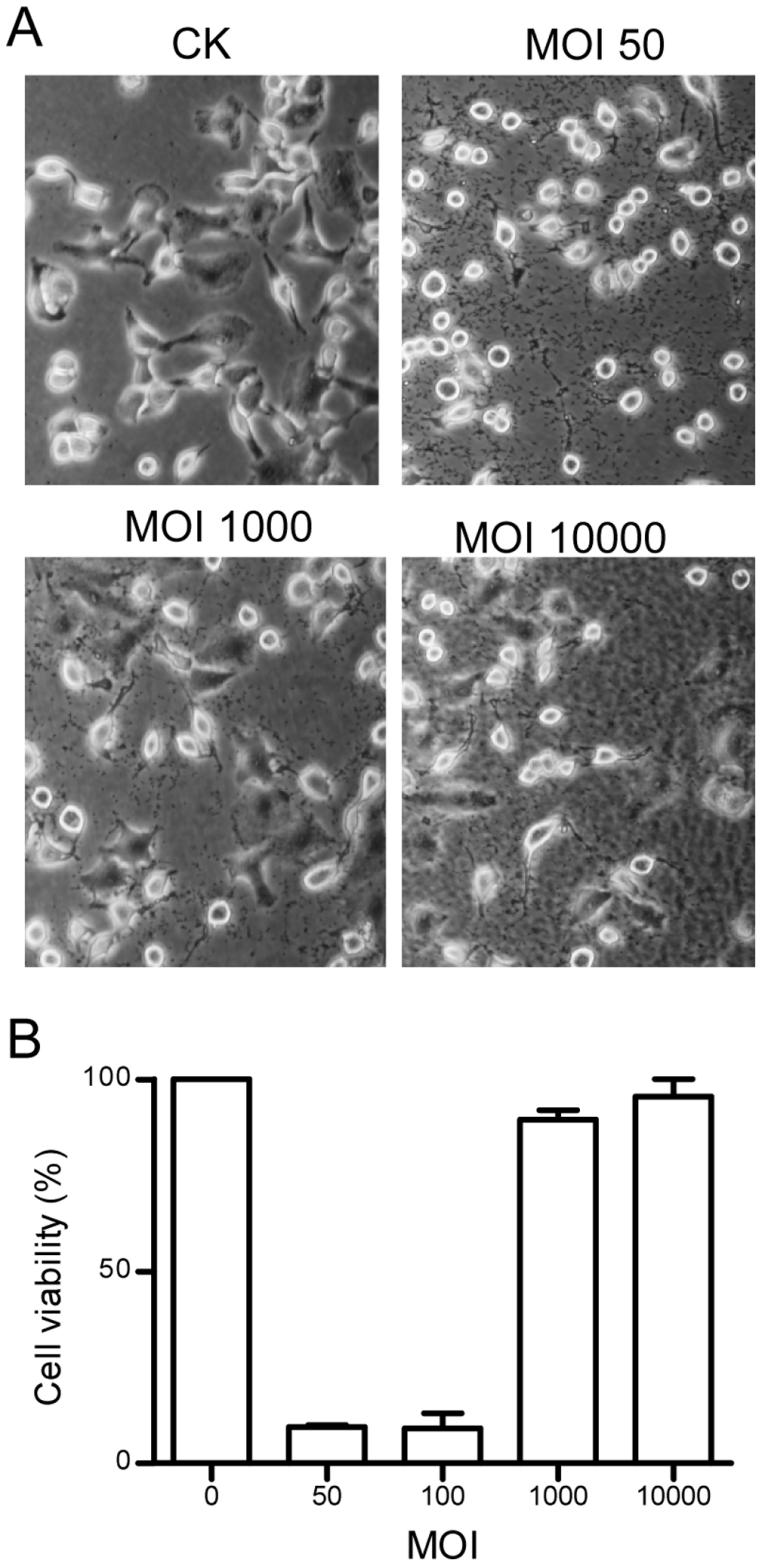
The cytotoxicity of *P. aeruginosa* was attenuated at high bacterial population density. (A) Microscope images of A549 cells infected with different dosages of bacterial cells. Control (CK) was A549 culture without bacterial infection. Photographs were taken under common microscope (10 × objective). (B) Quantitative analysis of A549 cell viability against different dosages of bacterial cells (PAO1) infection. A549 cell viability was measured by WST-1 test 4 h after inoculation. The experiment was repeated three times and the data shown are the means of 3 replicates with standard deviation.

In the process of optimizing conditions for assay of *P. aeruginosa* virulence, we noticed that the bacterial cytotoxicity might get lost when a high cell density inoculum was used to challenge the human lung cell line A549. The observation motivated us to investigate the dosage effect of *P. aeruginosa* inoculum on the survival rate of human cells. A serial dilution of *P. aeruginosa* PAO1 inoculum were prepared using DMEM medium and used to infect A549 cells. The results showed that the cytotoxicity of PAO1 was basically in reverse proportion to the cell density of the bacterial inoculum (Fig. 1). While hardly any livable A549 cells could be observed under microscope 4 h after infection with a low cell density bacterial inoculum of a multiplicity of infection (MOI) of 50, surprisingly, most A549 cells were remained alive when infected with bacterial cells at 1000 MOI or higher (Fig. 1A). Quantitative analysis confirmed that about 95% and 90% of A549 cells were killed when challenged with PAO1 at an MOI of 50 and 100, respectively (Fig. 1B), In contrast, only 5% - 1% of A549 cells were killed 4 h after inoculation with 1,000–10,000 MOI of PAO1 (Fig. 1B).

### *P. aeruginosa* Produces Virulence-inhibitory Molecule(s) at High Cell Density

**Figure 2:**
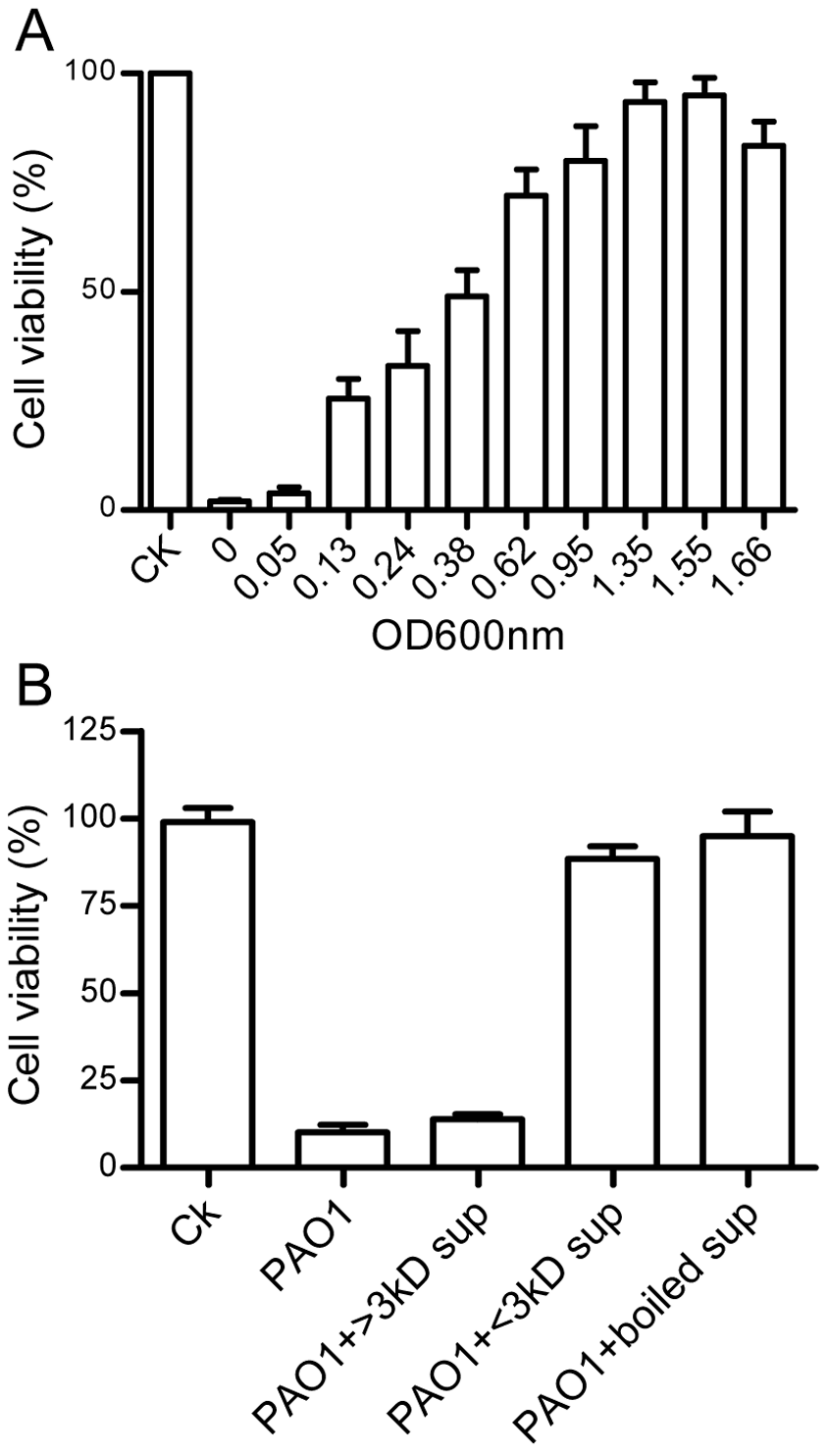
*P. aeruginosa* produces a small chemical inhibitor(s) in a cell density-dependent manner. (A) Virulence-inhibitory activity of strain PAO1 culture supernatants. The bacterial supernatants of different growth stages (OD_600_) were used as co-culture mediums when A549 was infected against PAO1 (MOI 50). Control (CK) is the normal A549 culture without bacterial infection. (B) The physical properties of the putative virulence inhibitor(s). PAO1 supernatants (sup) at OD_600_ = 1.6 were prepared as larger (>) and smaller (<) than 3 kD fractions respectively by using a Centricon-3 ultrafiltration device. A portion of PAO1 sup was boiled at 100°C for 10 min before bioassay. These sup preparations were used to prepare PAO1 inoculum and infect A549 cell lines as described above. The experiment was repeated three times and the data shown are the means of 3 replicates with standard deviation.

The above results suggest that *P. aeruginosa* PAO1 may produce inhibitory molecules at high cell density. To test this possibility, the supernatants were prepared from PAO1 cultures of different cell density and used to replace the DMEM medium to resuspend and dilute the PAO1 cells for inoculation. The results showed that addition of LB medium did not affect the cytotoxicity of PAO1, however, addition of the culture supernatants of different growth stages could attenuate the virulence of PAO1 in a bacterial cell-density-dependent manner (Fig. 2A). While the supernatants at OD_600_ = 0.05 had hardly any effect on bacterial virulence, the supernatants at OD_600_ = 1.35 and 1.55 almost completely blocked the bacterial virulence (Fig. 2A). Further experiments showed that the virulence-inhibiting activity of the bacterial supernatants remained unchanged by boiling (Fig. 2B), suggesting that the inhibitor is unlikely to be a protein molecule.

To estimate the molecular size of the putative inhibitor, the supernatants were fractionated by using a membrane filter with molecular weight cutoff of 3 kD. Bioassay results showed that that the inhibiting activity was detected only in the fraction containing the molecules less than 3 kD (Fig. 2B), indicating that *P. aeruginosa* PAO1 produces a virulence inhibitor(s) which is a small chemical molecule.

### Purification and Structural Characterization of the Putative Virulence Inhibitor

**Figure 3:**
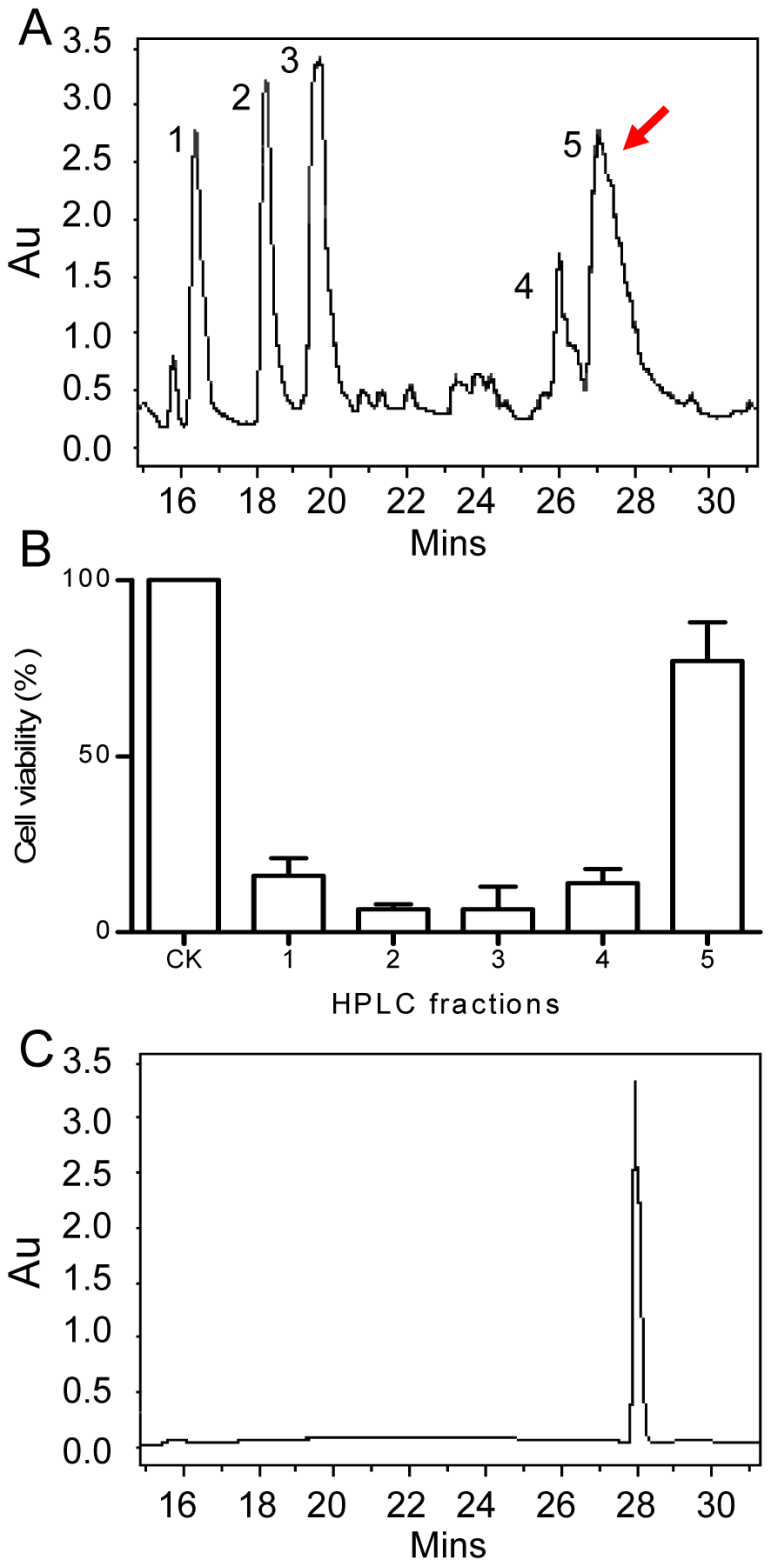
HPLC purification of the putative virulence inhibitor from *P. aeruginosa* culture supernatants. (A) HPLC separation of the active fractions obtained from Sephadex LH20 size-exclusion chromatography. (B) Bioassay to determine the virulence inhibitory activity of HPLC fractions shown in (A). The data shown are the means 3 replicates with standard deviation. (C) HPLC profile of the purified compound.

**Figure 4:**
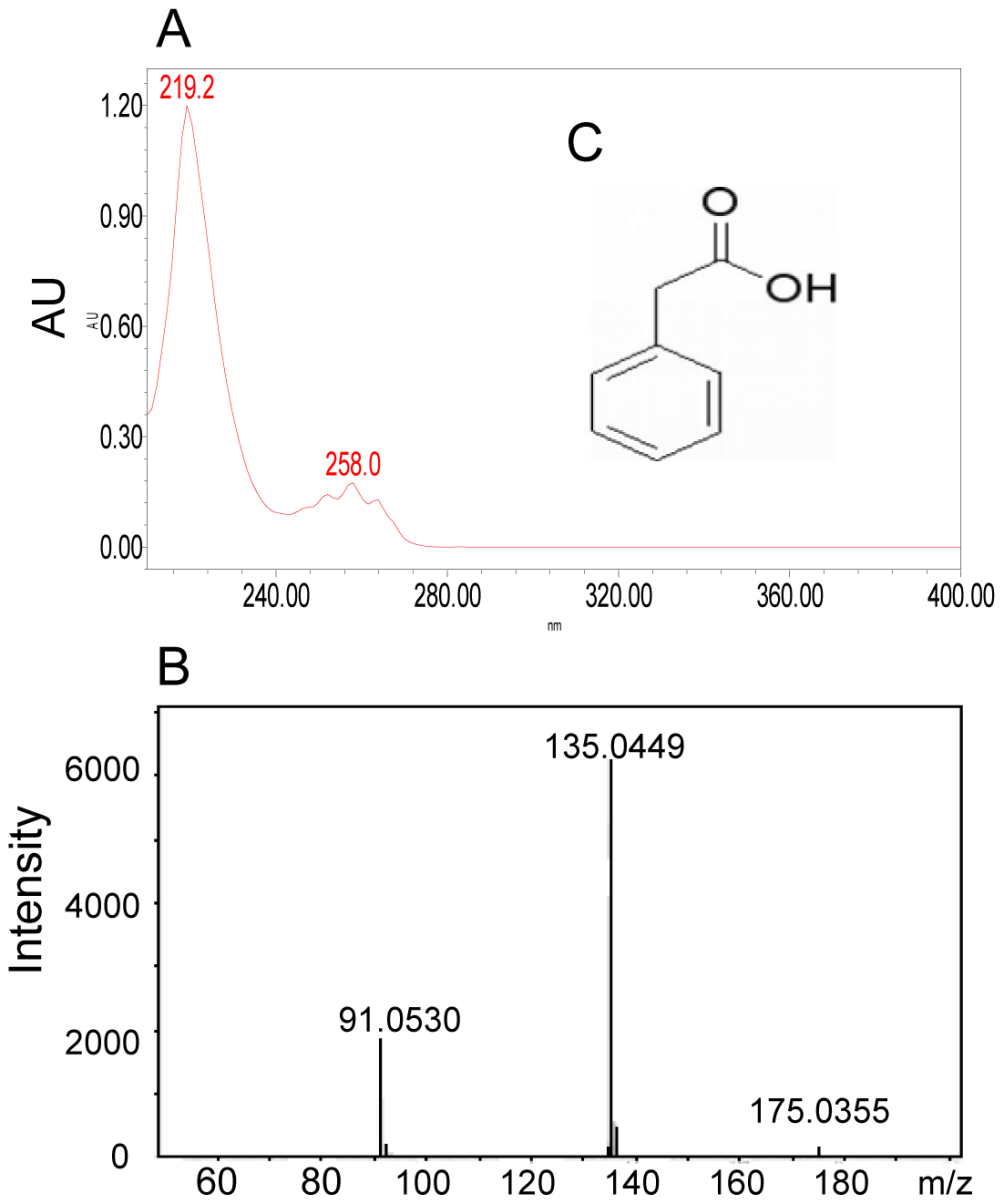
UV and Mass spectrometry analysis of the purified inhibitor. (A) UV spectrum. (B) High-resolution MS spectrum. (C) The predicted chemical structure of the purified chemical inhibitor phenylacetic acid.

The stationary-phase culture supernatants of PAO1 was extracted by ethyl acetate, and separated by Sephadex LH20 column chromatography. Active fractions were combined and separated further by high-performance liquid chromatography (HPLC) (Fig. 3A). Bioassay of the elutes led to identify a single peak (peak 5) with retention time at about 27 min which showed a strong protective activity against *P. aeruginosa* infection (Fig. 3A, 3B). The active fraction was further purified by HPLC (Fig. 3C), and about 5 mg of pure inhibitor was obtained from the 2 liters of PAO1 culture supernatants after evaporation of the solvent. UV spectrometry analysis showed that the purified virulence-inhibitor has two UV absorption peaks at 219.2 and 258.0 nM, respectively (Fig. 4A). In particular, the 258 nM peak is accompanied by typical shoulder peaks at right and left hand sides, suggesting a simple phenolic compound. High-resolution electrospray ionization mass spectrometry (ESI-MS) showed the m/z (M-H) of PAA (M-H) to be 135.0448 (Fig. 4B), suggesting a molecular formula of C_6_H_4_N_2_O_2_ (136.0273) or C_8_H_8_O_2_ (136.0524). _1_H NMR and _13_C NMR analysis showed that the inhibitor contains 8 carbons and 8 hydrogens (Table S3, Fig. S1, S2 in File S1). Taken together, we conclude that the purified inhibitor is phenylacetic acid (PAA) (chemical formula: C_8_H_8_O_2_; the calculated molecular weight  = 136.0524) (Fig. 4C).

Consistent with the results of structural analysis, synthetic PAA showed a similar HPLC and UV spectra (Fig. S3 in File S1), and comparable virulence-inhibitory activity against *P. aeriginosa* infection as the purified inhibitor (Fig. S4A in File S1). The results also showed that PAA at a concentration range from 10–10,000 µM did not have any visible effect on the growth of A549 cell line (Fig. S4B in File S1).

### The Protective Role of PAA on Host Cell Viability is Associated with its Activity on Bacterial Cells

**Figure 5:**
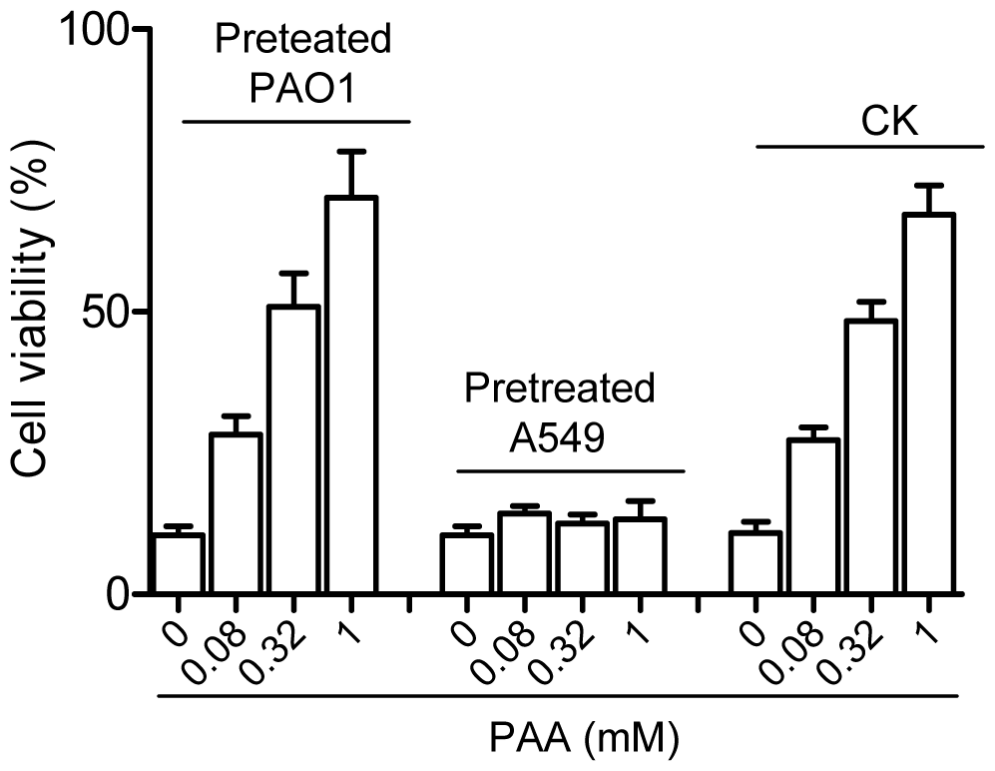
PAA protected A549 against PAO1 infection through its effect on bacteria. Prior to infection, PAO1 and A549 were treated respectively with different dosages of PAA for 2 h, then the pretreated PAO1 was used to infect normal A549, and compared with pretreated A549 and normal A549 infected against normal PAO1. All infections were last for 4 h at 37°C (MOI 50). Viability was measured by WST-1 test 4 h after inoculation. The experiment was repeated three times and the data are the means of 3 replicates with standard deviation and expressed as percentage over control.

To determine whether PAA reduces the bacterial cytotoxicity through activation of host cell defense mechanism or suppression of bacterial virulence or both, we treated PAO1 and A549 cells respectively with PAA for 2 h prior to cytotoxicity assay. The assay was divided into three groups: (1) normal A549 cells were inoculated with the PAA-treated PAO1 bacteria, (2) PAA-treated A549 cells were inoculated with normal PAO1 bacteria, and (3) normal A549 cells were inoculated with normal PAO1 bacteria and treated with various dosages of PAA as controls. The results showed that pretreatment of A549 with different amount of PAA before inoculation with untreated PAO1 had not shown any substantial protective activity on the cell viability of host cells (Fig. 5). In contrast, pretreatment of PAO1 with PAA before challenging A549 increased the A549 cell viability in a dosage-dependent manner, highly similar to the controls in which various amount of PAA were added to A549 shortly prior to infection with untreated PAO1 (Fig. 5). The results indicate that the protective activity of PAA on A549 viability is due to its effect on bacteria but not on host cells.

### Exogenous Addition of PAA Inhibits the Transcriptional Expression of T3SS Genes

To determine the scope of PAA influence on bacterial global gene expression profiles, we conducted microarray analysis of strain PAO1 treated with and without 1 mM PAA under T3SS-induction conditions (calcium depletion by addition of NTA to growth medium). The results showed that about 64 and 65 genes were down-regulated and up-regulated in *P. aeriginosa*, respectively, after treatment with 1 mM PAA (Table S4, Table S5 in File S1). The down-regulated genes can be grouped into the following 5 functional groups: (1) T3SS, (2) metabolism and catabolism, (3) transport, (4) regulators, and (5) hypothetical proteins (Table S4 in File S1); and the up-regulated genes belong to the following functional groups: (1) metabolism and catabolism, (2) transport, (3) regulators, and (4) hypothetical proteins (Table S5 in File S1). The data suggest that exogenous addition of PAA could generate a substantial impact on the bacterial genetic regulatory networks and physiology.

**Figure 6:**
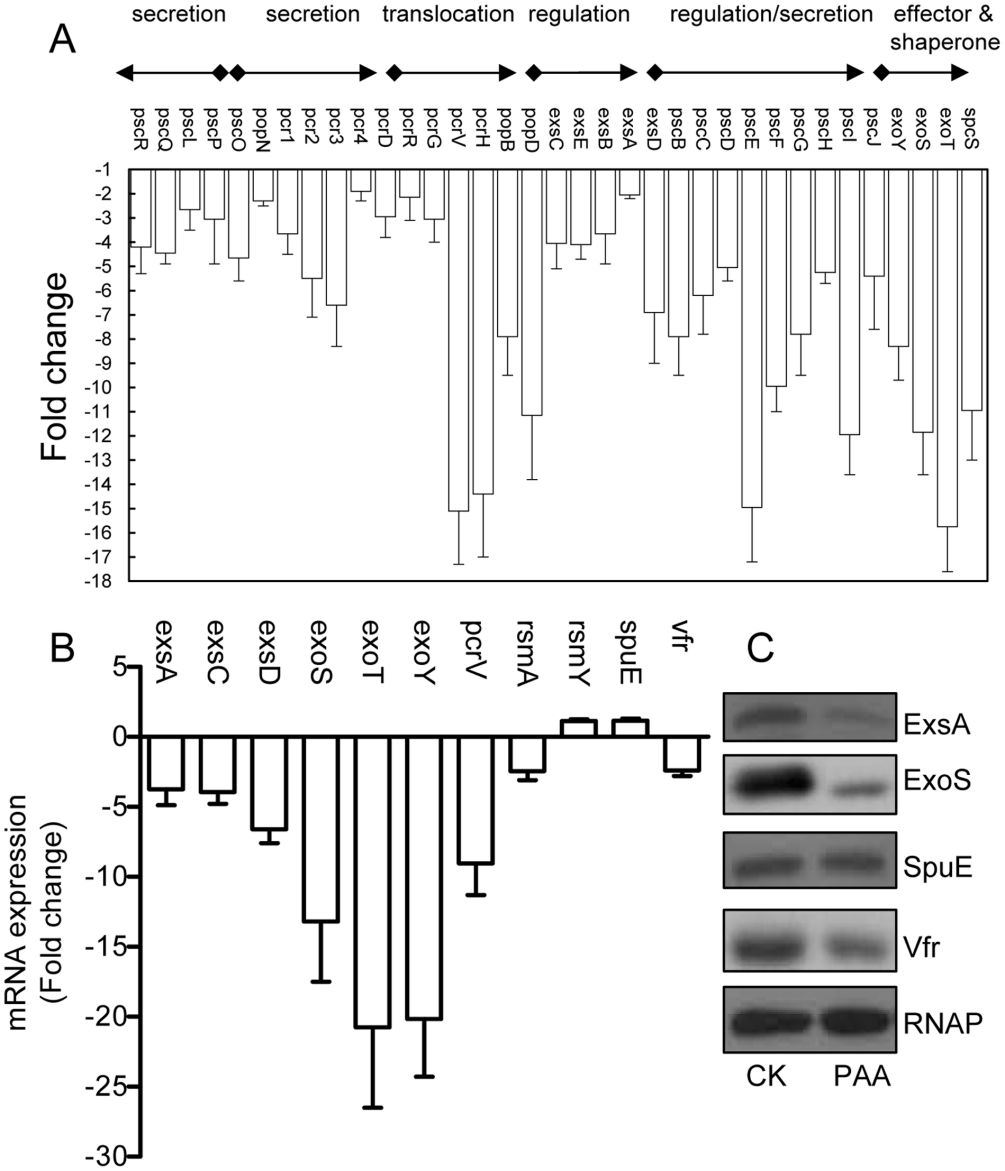
Effect of PAA on bacterial T3SS. (A) The transcriptional changes of T3SS genes in strain PAO1 treated with exogenous PAA compared to untreated PAO1. The experiment was repeated for two times. (B) Real-time PCR measurements of the mRNA levels of T3SS-related genes after treated with 1 mM PAA compared to the control without treatment. (C) Immunoblotting detection of T3SS-related proteins. Bacterial cultures were grown in LB medium supplemented with NTA as indicated. The extra-cellular proteins (ECP) and intra-cellular proteins (ICP) from strains PAO1 were separated by 10% SDS-PAGE. The proteins were transferred onto nitrocellulose membrane and blotted with antibodies.

Among the known virulence genes, the transcriptional expression of a total of 30 T3SS genes were significantly down-regulated (≥2-fold) in the PAA treated bacterial cells (Fig. 6A). The transcriptional expressions of three T3SS effector genes (*exoT*, *exoY*, *exoS*) were markedly decreased by 16.5, 9 and 12-fold, respectively (Fig. 6A). Translocation of the effectors is dependent on the proteins encoded by the *pcrGVHpopBD* operon. These proteins form a T3SS translocator complex, composed of a needle-tip complex (PcrV), translocons (PopB and PopD), and chaperones (PcrG and PcrH). PcrV mediates the folding and insertion of PopB/PopD in host plasmic membranes, where the assembled translocons form a translocation channel. Assembly of this complex and delivery of effectors through this machinery is tightly controlled by PcrV [23]. Significantly, *pcrV* transcription was decreased by about 15-fold by PAA treatment (Fig. 6A). Real time RT-PCR analysis of randomly selected T3SS genes, including *exsA, exsC, exsD, exoS, exoT, exoY* and *pcrV*, confirmed the negative regulatory role of PAA on the transcriptional expression of T3SS genes (Fig. 6B).

Among the known T3SS regulatory genes, we noticed that a few of them were also moderately affected (>1.2-fold) at the transcripts level, including *rsmA*, *vfr*, *algU*, *nirS*, and *cpdA* (Table S6 in File S1). Agreeable with the microarray data, real-time RT-PCR results showed that the transcripts level of *rsmA* and *vfr* were down-regulated by PAA treatment, while the spermidine transporter gene *spuE* and small RNA *rsmY* were moderately upregulated (Fig. 6B). The results were further confirmed by immunoablotting analysis which showed that the peptide levels of ExsA, ExoS and Vfr were substantially reduced by PAA treatment (Fig. 6C). As RsmA and Vfr are two important upstream regulators of *exsA*, the findings suggest that PAA may regulate T3SS genes through modulation of the T3SS up-stream signaling pathways.

### The Regulatory Role of PAA on T3SS Expression Requires a Functional RsmA

**Figure 7:**
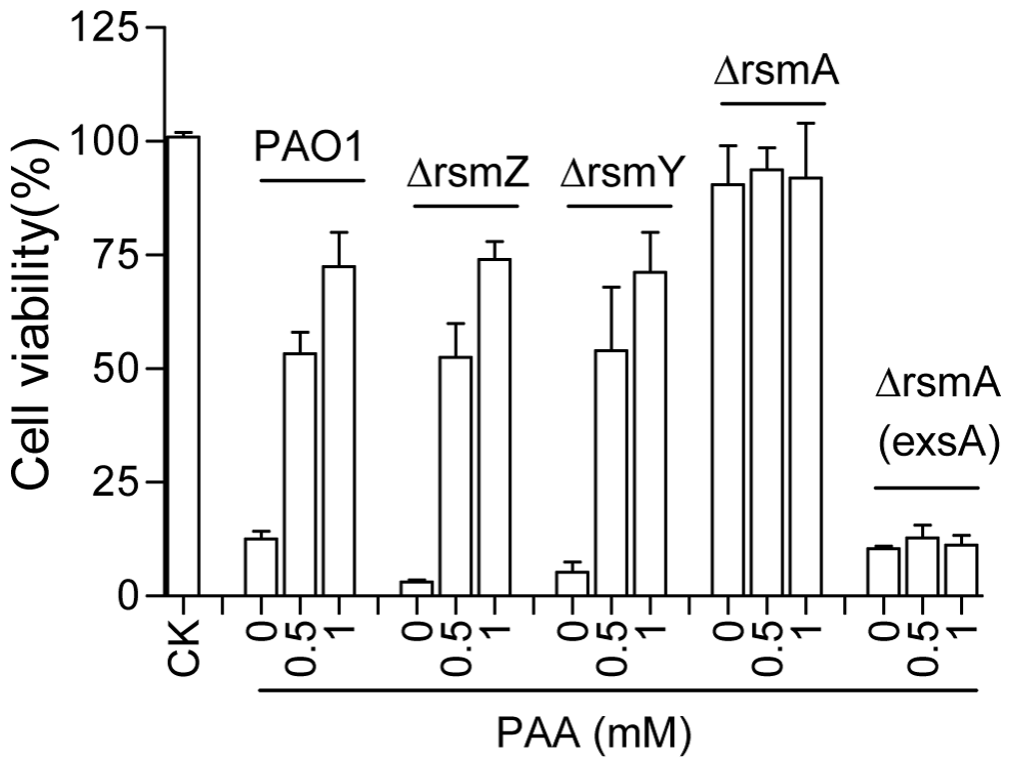
Protective effect of different dosages of PAA on A549 against wild type PAO1 and its derivatives. A549 without bacterial infection was used as control (CK). Viability was measured by WST-1 test 4 h after inoculation. The experiment was repeated three times and the data are the means of 3 replicates with standard deviation and expressed as percentage over control.

RsmA is a small RNA-binding protein which is responsible for the regulation of the key T3SS regulator ExsA, whereas RsmZ and RsmY are small RNAs which counteract the function of RsmA. To determine the separate role of each member of the RsmZYA system in PAA-mediated inhibition of T3SS, we generated the deletion mutants of *rsmA*, *rsmY* and *rsmZ*, respectively, using PAO1 as the parental strain and their virulence against cell line A549 were assayed in the presence or absence of PAA. Agreeable with the previous report [24], deletion of *rsmZ* or *rsmY* had no effect on bacterial virulence but null mutation of *rsmA* substantially attenuated the virulence (Fig. 7). Similar to the wild type strain PAO1, the virulences of the deletion mutants ΔrsmZ and ΔrsmY were inhibited by exogenous addition of PAA in a dosage-dependent manner, whereas the *rsmA* deletion mutant displayed a low cytotoxicity disregarding the presence or absence of PAA (Fig. 7). In contrast, *in trans* expression of the T3SS master regulator gene *exsA* in ΔrsmA made the strain highly virulent and indifferent to the added PAA (Fig. 7). The data suggest that a functional RsmA is required for the activity of PAA and confirm the previous speculation that PAA may regulate T3SS through modulation of the up-stream regulatory mechanisms.

## Discussion

A previous report showed that addition of the stationary-phase culture supernatants to exponential phase growing *P. aeruginosa* can inhibit T3SS expression [20], but the mechanism behind this observation remains unresolved. In this study, we showed that the cytotoxicity of *P. aeruginosa* is inversely proportional to its population density (Fig. 1, Fig. 2A). Bioassay-guided HPLC and spectrometry analyses showed that this population-density-dependent attenuation of virulence appears at least in part due to production and accumulation of PAA (Fig. 3; Fig. 4), a secondary metabolite derived from phenylalanine. It is estimated that the PAA level in the bacterial supernatants may be in the range between 74–184 µM, based on the assumption of 10–25% recovery rate after the multistep purification described in this study. The results in Fig. 5 showed that exogenous addition of 80 µM PAA could substantially reduce the bacterial virulence, indicating the physiological relevance of PAA in modulation of bacterial virulence. We also noticed the dosage dependent pattern of PAA in affecting bacterial virulence, increasing PAA concentration from 80 µM to 320 and 1000 µM (by 4 and 12.5 times, respectively) further reduced the bacterial virulence by about 2.2- and 3.3-fold, respectively (Fig. 5). To evaluate the full impact of PAA on bacterial virulence and physiology, we therefore used 1 mM PAA in microarray analysis to unveil the PAA influenced genes. The results showed that PAA specifically down-regulates the T3SS gene expression among various known virulence genes of *P. aeruginosa* (Table S4 in File S1; Fig. 6). T3SS is the major virulence determinant of bacterial early infection and acute cytotoxicity [3]–[8]. Identification of PAA as a T3SS inhibitor provides a molecular basis to the early observation that the expression level of *P. aerugionsa* T3SS genes drops drastically at the stationary growth phase [18].

It was speculated that the T3SS-inhibitory molecule in the stationary-phase culture supernatants of *P. aeruginosa* could be IAA [20], but our bioassay-guided HPLC separation of bacterial supernatants showed that PAA rather than IAA is the major T3SS inhibitory molecule in the bacterial culture supernatants. PAA is believed to be derived from phenyalanine [25], but the gene encoding PAA biosynthesis has not yet been reported. Interestingly, it was shown previously that PAA in *Azospirillum brasilense* is synthesized by indole-3-pyruvate decarboxylase [25], which is the key enzyme for IAA biosynthesis. However, a Blast search did not reveal an obvious homologue of indole-3-pyruvate decarboxylase in the genome of *P. aeruginosa*. At this stage, we could not rule out that IAA may be produced as a minor component by *P. aeruginosa*. Consistent with our results, the previous study failed to identify the detectable amount of IAA in the culture supernatants of *P. aeruginosa* [20]. In addition, we found that the T3SS inhibitory activity of IAA was inferior than PAA (Fig. S5 in File S1).

Microarray analysis showed that exogenous addition of PAA decreased the transcriptional expression of several regulators implicated in T3SS regulation, including ExsA, RsmA and Vfr (Fig. 6B, 6C). Among them, ExsA is the master regulator which directly control the transcriptional expression of T3SS genes by binding to their promoters [11], [12], and RsmA is an RNA binding protein that functions at the upstream of ExsA and plays a positive role in regulation of T3SS in *P. aeruginosa* [26]. Interestingly, PAA also upregulates the transcripts level of *rsmY* and *rsmZ* (Fig. 6), which are small RNAs positively controlled by the GacS/GacA two component system [26], [27]. The two small RNAs are negative regulators in T3SS modulation as they act by binding to and thus inactivating RsmA [26]. The similar regulatory pattern of PAA on ExsA and RsmA transcriptional expression suggest that PAA may influence ExsA level through regulation of *rsmA/rsmZ/rsmY* transcription. Consistent with this view, we found that deletion of *rsmA* abolished the inhibitory effect of PAA and the strain constitutively expressing *exsA* maintained full virulence with or without PAA (Fig. 7).

It has been a long standing observation that metabolites are associated with the regulation of T3SS gene expression in *P. aeruginosa* [20], [28], [29], [30]. A range of metabolic signals have been implicated in the regulation of T3SS including pyruvate imbalance [29], and tryptohan metabolites [20], but the chemical nature of the putative signal molecules has not yet been determined. Identification of PAA as a key metabolic signal involved in the arrest of the positive activation loop of the *P. aeruginosa* T3SS may provide a useful platform to probe the detailed molecular mechanisms of the metabolites mediated T3SS gene expression arrest, which may further contribute to design and developing novel strategies in control and prevention of bacterial virulence.

## Supporting Information

File S1 **Figure S1.** The _1_H NMR spectrum of PAA. **Figure S2.** The _12_C NMR spectrum of PAA. **Figure S3.** HPLC profile and UV spectrometry analysis of synthetic PAA. (A) HPLC of synthetic PAA, (B) UV spectrum of synthetic PAA (C) HPLC of purified PAA. (D) UV spectrum of purified PAA. **Figure S4.** Comparative analysis of synthetic and purified PAA. (A) Different dosages of synthetic PAA were used to protect A549 against PAO1 infection, and compared with purified PAA. (B) The effect of different dosages of synthetic PAA on growth of A549. Viability was measured by WST-1 test. Data are expressed as the percentage of internal control for each cell type measured 12 h after cell seeding (time 0) and are the mean ± SEM of 3 replicates. The experiment was repeated at least three times. **Figure S5.** Protection effect of different dosages of PAA and IAA against PAO1 infection. Control is normal A549 without bacterial infection. Viability was measured by WST-1 test. The experiment was repeated three times and the data shown are the means of 3 replicates with standard deviation and expressed as the percentage of control. **Figure S6.** PAA affects PAO1 virulence by down regulation of T3SS, not by inhibition of bacterial growth. (A) Effect of PAA on T3SS gene expression. The *exsCEBA* Promoter- directed β-galactosidase activity was determined 4 h after culture of the bacterial cells with different dosages of PAA. (B) Effect of PAA on bacterial growth. OD_600_ of bacterial cells (PAO1) was measured 4 h after culture of the bacterial cells with different dosages of PAA. **Table S1.** Strains and plasmids used in this study. **Table S2.** Primers for real time PCR. **Table S3.** 1H and 13C NMR of PAA in Methanol-d4. **Table S4.** Down-regulated genes in strain PAO1 after treatment with PAA. **Table S5.** Up-regulated genes in strain PAO1 after treatment with PAA. **Table S6.** T3SS regulator change after PAA treatment.
(PDF)
