# Supplementary material for: Pseudomonas aeruginosa Cytotoxicity Is Attenuated at High Cell Density and Associated with the Accumulation of Phenylacetic Acid
Source: PLoS One. 2013 Mar 29;8(3):e60187. doi: 10.1371/journal.pone.0060187 (PMC3612096; doi:10.1371/journal.pone.0060187)
Supplement: File S1 — Figure S1. The 1H NMR spectrum of PAA. Figure S2. The 12C NMR spectrum of PAA. Figure S3. HPLC profile and UV spectrometry analysis of synthetic PAA. (A) HPLC of synthetic PAA, (B) UV spectrum of synthetic PAA (C) HPLC of purified PAA. (D) UV spectrum of purified PAA. Figure S4. Comparative analysis of synthetic and purified PAA. (A) Different dosages of synthetic PAA were used to protect A549 against PAO1 infection, and compared with purified PAA. (B) The effect of different dosages of synthetic PAA on growth of A549. Viability was measured by WST-1 test. Data are expressed as the percentage of internal control for each cell type measured 12 h after cell seeding (time 0) and are the mean ± SEM of 3 replicates. The experiment was repeated at least three times. Figure S5. Protection effect of different dosages of PAA and IAA against PAO1 infection. Control is normal A549 without bacterial infection. Viability was measured by WST-1 test. The experiment was repeated three times and the data shown are the means of 3 replicates with standard deviation and expressed as the percentage of control. Figure S6. PAA affects PAO1 virulence by down regulation of T3SS, not by inhibition of bacterial growth. (A) Effect of PAA on T3SS gene expression. The exsCEBA Promoter- directed β-galactosidase activity was determined 4 h after culture of the bacterial cells with different dosages of PAA. (B) Effect of PAA on bacterial growth. OD600 of bacterial cells (PAO1) was measured 4 h after culture of the bacterial cells with different dosages of PAA. Table S1. Strains and plasmids used in this study. Table S2. Primers for real time PCR. Table S3. 1H and 13C NMR of PAA in Methanol-d4. Table S4. Down-regulated genes in strain PAO1 after treatment with PAA. Table S5. Up-regulated genes in strain PAO1 after treatment with PAA. Table S6. T3SS regulator change after PAA treatment. (PDF) [file pone.0060187.s001.pdf]

SUPPLEMENTARY INFORMATION

***Pseudomonas aeruginosa* cytotoxicity is attenuated at high cell density and associated with the accumulation of phenylacetic acid**

Jianhe Wang et al
Institute of Molecular and Cell Biology, 61 Biopolis Drive, Singapore 138673

Content:

Table S1. Strains and plasmids used in this study
Table S2. Primers for real time PCR
Table S3. $_{1}$H and $_{13}$C NMR of PAA in Methanol-d_4
Table S4. Down-regulated genes in strain PAO1 after treatment with PAA
Table S5. Up-regulated genes in strain PAO1 after treatment with PAA
Table S6. T3SS regulator change after PAA treatment
Fig S1. The $_{1}$H NMR spectrum of PAA
Fig S2. The $_{12}$C NMR spectrum of PAA
Fig S3. HPLC profile and UV spectrometry analysis of synthetic PAA
Fig S4. Comparative analysis of synthetic and purified PAA
Fig S5. Protection effect of PAA and IAA against PAO1
Fig S6. PAA affects PAO1 virulence by down-regulation of T3SS

Table S1. Strains and plasmids used in this study

Strains or plasmid	Description	Source or reference
P. aeruginosa		
PAO1	Prototrophic laboratory strain	Laboratory collection
PAO1pClacZ	*lacZ* fused to the *exsCEBA* promoter and integrated at the *attB* site of the PAO1 chromosome	Laboratory collection
PAO1 pClacZΔrsmZ	A *rsmZ* deletion mutant of PAO1pClacZ	Laboratory collection
PAO1 pClacZΔrsmY	A *rsmY* deletion mutant of PAO1pClacZ	Laboratory collection
PAO1 pClacZΔrsmA	A *rsmA* deletion mutant of PAO1pClacZ	Laboratory collection
Plasmid		
pDSK-exsA	ExsA overexpression plasmid	Laboratory collection

Table S2. Primers for real time PCR

Name	Primer	start at	stop at	Tm
PAO1 RT-qPCR				
rsmA-F	TGGGTGTCAAAGGGAACCA	68	86	58
rsmA-R	TGGTAAATTTCCTCCCGGTGTA	146	125	59
rsmY-F	TCAGGACATTGCGCAGGAA	2	20	60
rsmY-R	TTTGCAGACCTCTATCCTGACATC	100	77	58
vfr-F	GACGGCCGCGAAATGA	169	184	59
vfr-R	CCCAGCTCGCCGAAGA	227	212	58
exsA-F	CATGGAGGCGGGCTTTT	720	736	58
exsA-R	GCGTGCAGCCGAAACG	787	772	60
exsC-F	CATTGGCACCGTTTCGATCT	232	251	60
exsC-R	CTGCGCATACAACTGGACCTT	300	280	59
spuE-F	GCGACCAGCCGATCGA	446	461	59
spuE-R	TGGCGAGCTTCTTCATGTTCT	508	488	59
trpB+A-F	TGCAACAGGAGTCGAAAGCAT	1187	1207	59
trpB+A-R	CGCGGTTTTCCTGCTTGA	1254	1237	60
exsD+-F	CCAGGCACAGGATCACTTCTACT	798	820	58
exsD+-R	CGCTCAACAGATGATCCATGA	883	863	58
proC-F	CTTCGAAGCACTGGTGGAG	744	762	59
proC-R	TTATTGGCCAAGCTGTTCG	804	822	59

Table S3. ^{1}H and ^{13}C NMR of PAA in Methanol-d_4

Position*	δH (ppm)	δC (ppm)
1	-	175.6
2	3.6, s	42.0
3	-	136.1
4 (8)	7.3, m	130.4
5 (7)		129.5
6		127.9

*The carbon and hydrogen positions are labelled below:

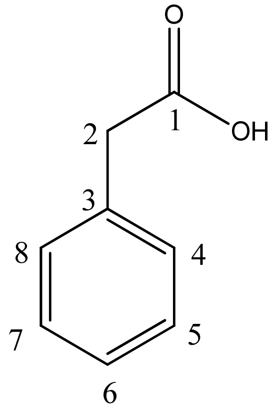

Table S4. Down-regulated genes in strain PAO1 after treatment with PAA (1.5 fold)*

Public ID	Gene Name	change fold	Product Name
T3SS			
PA0044	*exoT*	-16±2.3	exoenzyme T
PA1692		-4.1±0.6	probable translocation protein in type III secretion
PA1693	*pscR*	-4.4±1.2	translocation protein in type III secretion
PA1694	*pscQ*	-4.0±0.5	translocation protein in type III secretion
PA1695	*pscP*	-2.9±0.5	translocation protein in type III secretion
PA1696	*pscO*	-5.3±0.8	translocation protein in type III secretion
PA1697		-2.3±1.0	ATP synthase in type III secretion system
PA1698	*popN*	-2.0±0.2	Type III secretion outer membrane protein PopN precursor
PA1699	*pcr1*	-3.5±1.0	Pcr1
PA1700	*pcr2*	-5.3±1.7	Pcr2
PA1701	*pcr3*	-6.5±1.8	Pcr3
PA1702	*pcr4*	-1.6±0.3	Pcr4
PA1703	*pcrD*	-2.6±0.7	type III secretory apparatus protein PcrD
PA1704	*pcrR*	-1.5±1.2	transcriptional regulator protein PcrR
PA1705	*pcrG*	-3.4±1.1	regulator in type III secretion
PA1706	*pcrV*	-15±2.3	type III secretion protein PcrV
PA1707	*pcrH*	-14±2.1	regulatory protein PcrH
PA1708	*popB*	-8.2±1.3	translocator protein PopB
PA1709	*popD*	-11±2.3	Translocator outer membrane protein PopD precursor
PA1710	*exsC*	-3.7±1.2	ExsC, exoenzyme S synthesis protein C precursor.
PA1711	*exsE*	-3.8±0.4	ExsE
PA1712	*exsB*	-3.5±1.2	exoenzyme S synthesis protein B
PA1713	*exsA*	-1.9±0.1	transcriptional regulator ExsA
PA1714	*exsD*	-7.1±2.1	ExsD
PA1715	*pscB*	-6.1±1.3	Type III export apparatus protein
PA1716	*pscC*	-5.6±1.2	Type III secretion outer membrane protein PscC precursor
PA1717	*pscD*	-4.1±0.4	type III export protein PscD
PA1718	*pscE*	-14±2.0	type III export protein PscE
PA1719	*pscF*	-8.9±1.0	type III export protein PscF
PA1720	*pscG*	-8.1±1.5	type III export protein PscG
PA1721	*pscH*	-4.5±0.4	type III export protein PscH
PA1722	*pscI*	-11±1.2	type III export protein PscI
PA1723	*pscJ*	-4.1±1.5	type III export protein PscJ
PA1725	*pscL*	-2.0±0.5	type III export protein PscL
PA2191	*exoY*	-7.8±1.1	adenylate cyclase ExoY
PA3841	*exoS*	-11±1.2	exoenzyme S
PA3842	*spcS*	-11±1.1	specific Pseudomonas chaperone for ExoS, SpcS
Metabolism and catabolism			
PA0743		-1.5±0.3	probable 3-hydroxyisobutyrate dehydrogenase

PA0795	*prpC*	-1.7±1.0	citrate synthase 2
PA2003	*bdhA*	-2.3±1.1	3-hydroxybutyrate dehydrogenase
PA3584	*glpD*	-3.0±0.4	glycerol-3-phosphate dehydrogenase
PA5353	*glcF*	-1.5±0.5	glycolate oxidase subunit GlcF
PA5354	*glcE*	-1.7±0.6	glycolate oxidase subunit GlcE
PA5355	*glcD*	-1.5±1.0	glycolate oxidase subunit GlcD
PA5372	*betA*	-2.0±0.2	choline dehydrogenase
PA5373	*betB*	-1.8±0.1	betaine aldehyde dehydrogenase
Transport			
PA0280	*cysA*	-2.1±0.5	sulfate transport protein CysA
PA0958	*oprD*	-2.8±1.0	Basic amino acid, basic peptide and imipenem outer membrane porin OprD precursor
PA2329		-3.8±0.3	probable ATP-binding component of ABC transporter
PA3581	*glpF*	-2.1±0.5	glycerol uptake facilitator protein
PA3676		-1.6±0.3	probable Resistance-Nodulation-Cell Division (RND) efflux transporter
Regulators			
PA4296	*pprB*	-1.8±0.5	two-component response regulator, PprB
PA5374	*betI*	-2.4±0.6	transcriptional regulator BetI
PA0652	*vfr*	-1.3±0.2	transcriptional regulator Vfr
PA0905	*rsmA*	-1.5±0.15	RsmA, regulator of secondary metabolites
PA0760	*algU*	-1.2±0.2	sigma factor AlgU
PA4969	*cpdA*	-1.2±0.06	Cyclic AMP (cAMP) Phosphodiesterase, CpdA
**Others**			
PA3348		-1.5±0.1	probable chemotaxis protein methyltransferase
PA4306	*flp*	-1.6±0.3	Type IVb pilin, Flp
PA4309	*pctA*	-1.5±0.6	chemotactic transducer PctA
PA3723		-1.5±1.0	probable FMN oxidoreductase
Hypothetical proteins			
PA1216		-1.5±0.3	hypothetical protein
PA1559		-1.5±0.1	hypothetical protein
PA3843		-7.3±0.9	hypothetical protein
PA4782		-1.5±0.2	hypothetical protein
PA5352		-1.9±0.3	conserved hypothetical protein
PA5509		-1.5±0.5	hypothetical protein

Table S5. Up-regulated genes in strain PAO1 after treatment with PAA (1.5 fold)*

Public ID	Gene Name	Change fold	Product Name
Metabolism and catabolism			
PA0835	*pta*	1.7±1	phosphate acetyltransferase
PA2193	*hcnA*	2.1±0.1	hydrogen cyanide synthase HcnA
PA2194	*hcnB*	1.9±0.3	hydrogen cyanide synthase HcnB
PA2323		1.5±0.5	probable glyceraldehyde-3-phosphate dehydrogenase
PA3870	*moaA1*	2.1±0.2	molybdopterin biosynthetic protein A1
PA3872	*narI*	3.1±1	respiratory nitrate reductase gamma chain
PA3873	*narJ*	3.6±0.8	respiratory nitrate reductase delta chain
PA3874	*narH*	5.2±0.9	respiratory nitrate reductase beta chain
PA3875	*narG*	12.3±2	respiratory nitrate reductase alpha chain
PA3914	*moeA1*	3.0±0.1	molybdenum cofactor biosynthetic protein A1
PA3915	*moaB1*	9.9±0.2	molybdopterin biosynthetic protein B1
PA4125	*hpcD*	1.5±0.1	5-carboxymethyl-2-hydroxymuconate isomerase
PA4854	*purH*	1.6±0.8	phosphoribosylaminoimidazolecarboxamide formyltransferase
PA5297	*poxB*	1.8±0.3	pyruvate dehydrogenase (cytochrome)
PA5559	*atpE*	1.5±0.2	atp synthase C chain
Transport			
PA3188		1.6±0.3	probable permease of ABC sugar transporter
PA3877	*narK1*	44.6±3	nitrite extrusion protein 1
PA3876	*narK2*	24.2±5.1	nitrite extrusion protein 2
PA0162	*opdC*	4.7±2	histidine porin OpdC
PA5230		3.3±0.1	probable permease of ABC transporter
Regulators			
PA3458		4.8±1.1	probable transcriptional regulator
PA1196		3.4±1.2	probable transcriptional regulator
PA0403	*pyrR*	1.5±0.1	transcriptional regulator PyrR
PA0519	*nirS*	2.2±0.1	nitrite reductase precursor
PA4856	*retS*	1.2±0.08	RetS (Regulator of Exopolysaccharide and Type III)
Others			
PA3221	*csaA*	1.5±0.3	CsaA protein
PA4276	*secE*	1.5±0.5	secretion protein SecE
PA3871		2.9±0.6	probable peptidyl-prolyl cis-trans isomerase, PpiC-type
PA4498		1.6±1	probable metallopeptidase
PA3894		2.1±0.3	probable outer membrane protein precursor
Hypothetical proteins			
PA0616		1.5±0.4	hypothetical protein
PA0171		1.7±0.6	hypothetical protein
PA3575		6.5±0.8	hypothetical protein
PA5482		2.3±1.0	hypothetical protein
PA3602		2.0±0.6	conserved hypothetical protein

PA3893	1.6±0.1	conserved hypothetical protein
PA4317	1.5±0.3	hypothetical protein
PA5568	1.5±0.6	conserved hypothetical protein
PA1897	1.5±0.1	hypothetical protein
PA1747	32.8±5	hypothetical protein
PA0160	24.1±0.3	hypothetical protein
PA0161	8.2±4	hypothetical protein
PA3205	6.3±0.6	hypothetical protein
PA5232	4.9±2	conserved hypothetical protein
PA1673	4.2±1.2	hypothetical protein
PA1789	4.1±0.3	hypothetical protein
PA3613	3.3±1.1	hypothetical protein
PA0200	2.3±0.5	hypothetical protein
PA3309	2.1±0.1	conserved hypothetical protein
PA3779	2.1±0.6	hypothetical protein
PA4739	2.0±0.4	conserved hypothetical protein
PA1197	2.0±0.5	hypothetical protein
PA4738	1.8±0.2	conserved hypothetical protein
PA4611	1.8±0.3	hypothetical protein
PA0911	1.8±0.5	hypothetical protein
PA1658	1.8±0.3	conserved hypothetical protein
PA0170	1.7±1.0	hypothetical protein
PA3911	1.7±0.8	conserved hypothetical protein
PA3054	1.6±1.0	hypothetical protein
PA0201	1.6±0.5	hypothetical protein
PA1657	1.5±0.2	conserved hypothetical protein
PA1404	1.5±0.5	hypothetical protein
PA2747	1.5±0.1	hypothetical protein
PA2171	1.5±0.2	hypothetical protein

Table S6. T3SS regulatory gene changes after PAA treatment

Public ID	Gene Name	Change fold	Product Name
PA0652	*vfr*	-1.3±0.2	transcriptional regulator Vfr
PA0905	*rsmA*	-1.5±0.15	RsmA, regulator of secondary metabolites
PA0762	*algU*	-1.2±0.2	sigma factor AlgU
PA0763	*mucA*	-1.1±0.5	anti-sigma factor MucA
PA5261	*algR*	-0.2±0.1	alginate biosynthesis regulatory protein AlgR
PA3622	*rpoS*	-0.1±0.05	sigma factor RpoS
PA0612	*ptrB*	0.4±0.2	repressor, PtrB
PA2808	*ptrA*	0.0±0.3	two-component response repressor, PtrA
PA4969	*cpdA*	-1.2±0.07	Cyclic AMP (cAMP) Phosphodiesterase, CpdA
PA3217	*cyaB*	1.1±0.1	CyaB
PA1822	*fimL*	1.0±0.02	hypothetical protein
PA3192	*gltR*	-0.4±0.5	two-component response regulator GltR
PA0519	*nirS*	2.2±0.1	nitrite reductase precursor
PA0928	*gacS*	0.0±0.01	sensor/response regulator hybrid
PA2586	*gacA*	-0.2±0.02	response regulator GacA
PA5332	*crc*	0.0±0.1	catabolite repression control protein
PA0035	*trpA*	0.1±0.5	tryptophan synthase alpha chain
PA0036	*trpB*	1.0±0.02	tryptophan synthase beta chain
PA4856	*retS*	1.2±0.08	RetS (Regulator of Exopolysaccharide and Type III)
PA0913	*mgtE*	0.1±0.2	probable Mg transporter MgtE
PA1452	*flhA*	-1.0±0.1	flagellar biosynthesis protein FlhA

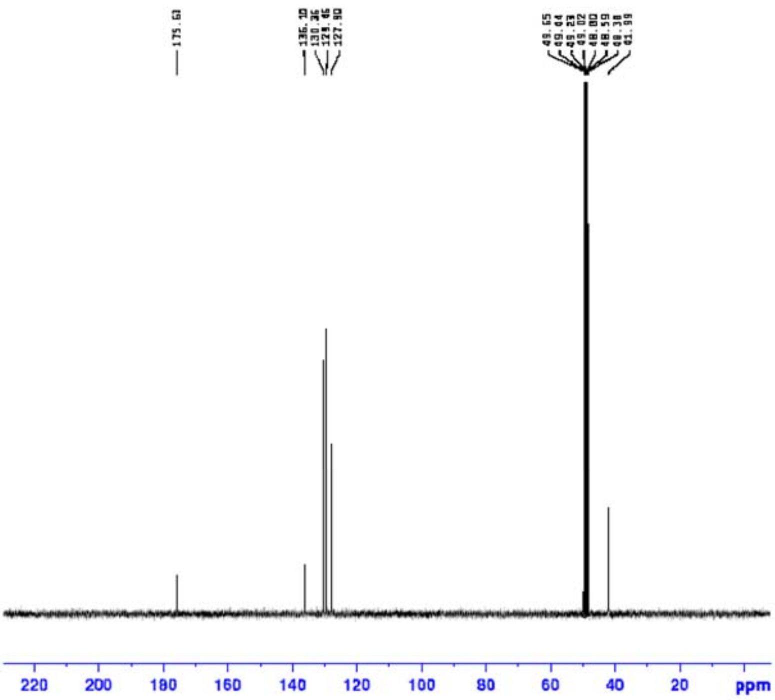
175.63
136.10
130.36
129.46
127.90
49.65
49.44
49.23
49.02
48.80
48.59
48.38
41.99
220
200
180
160
140
120
100
80
60
40
20
ppm

Fig S1.

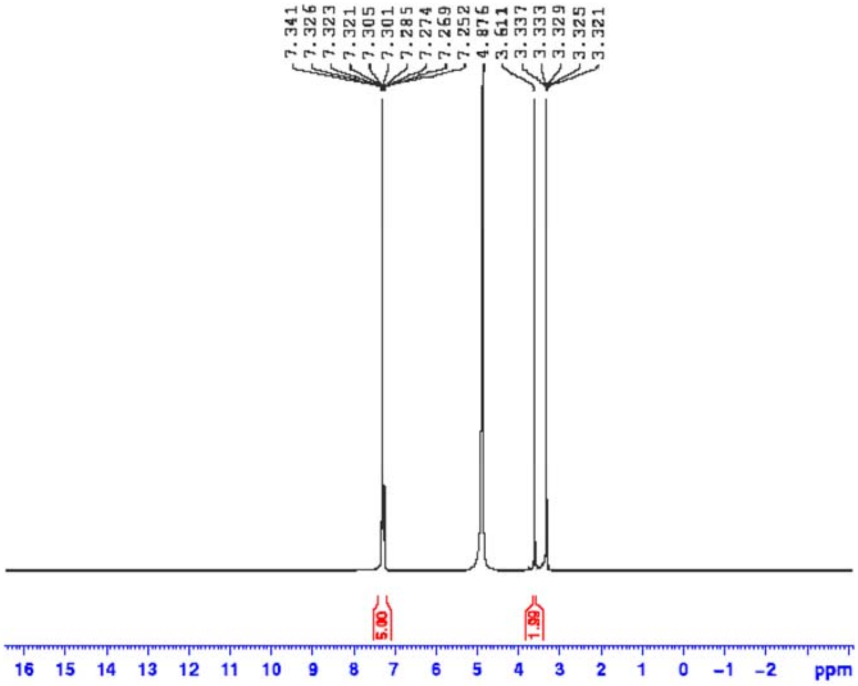
7.341
7.326
7.323
7.321
7.305
7.301
7.285
7.274
7.269
7.252
4.876
3.611
3.337
3.333
3.329
3.325
3.321
5.00
1.99
16 15 14 13 12 11 10 9 8 7 6 5 4 3 2 1 0 −1 −2 ppm

Fig S2.

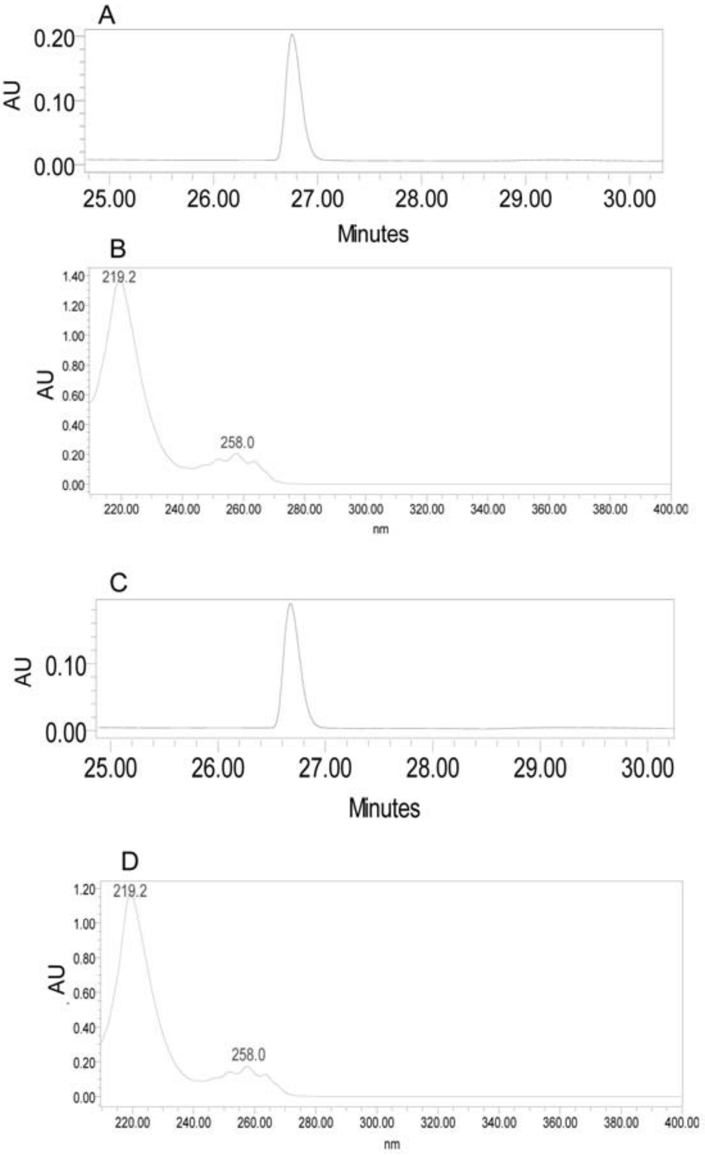
A
0.20
AU
0.10
0.00
25.00
26.00
27.00
28.00
29.00
30.00
Minutes
B
219.2
258.0
AU
nm
C
AU
0.10
0.00
Minutes
D
219.2
258.0
AU
nm

Fig S3.

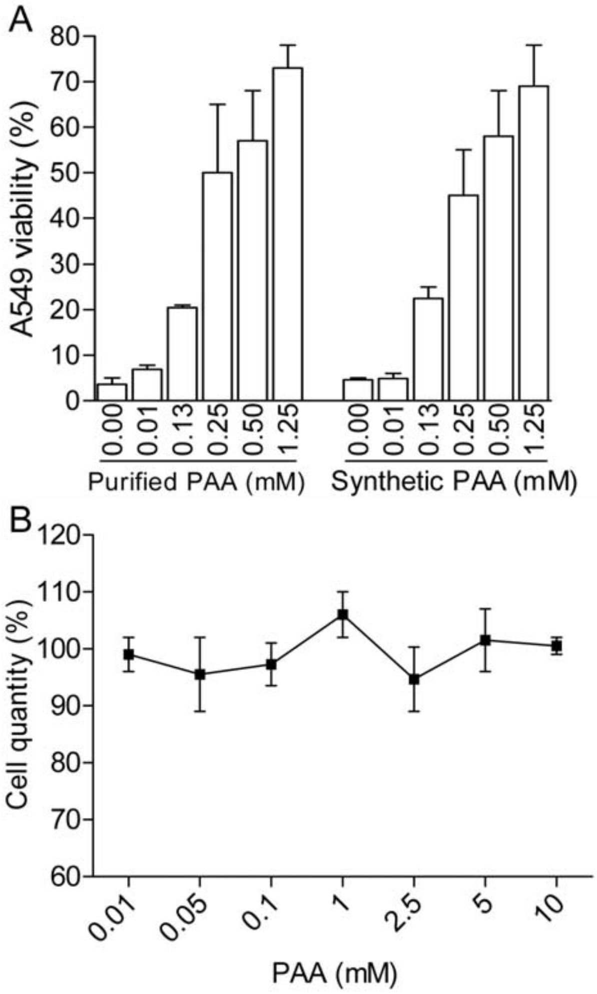
A
B
A549 viability (%)
0
10
20
30
40
50
60
70
80
0.00
0.01
0.13
0.25
0.50
1.25
Purified PAA (mM)
Synthetic PAA (mM)
Cell quantity (%)
60
70
80
90
100
110
120
0.01
0.05
0.1
1
2.5
5
10
PAA (mM)

Fig S4.

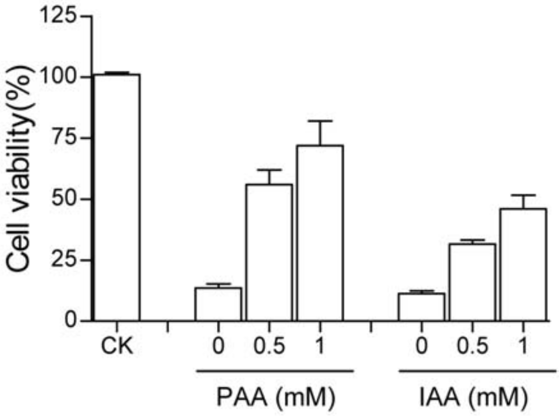

Fig S5.

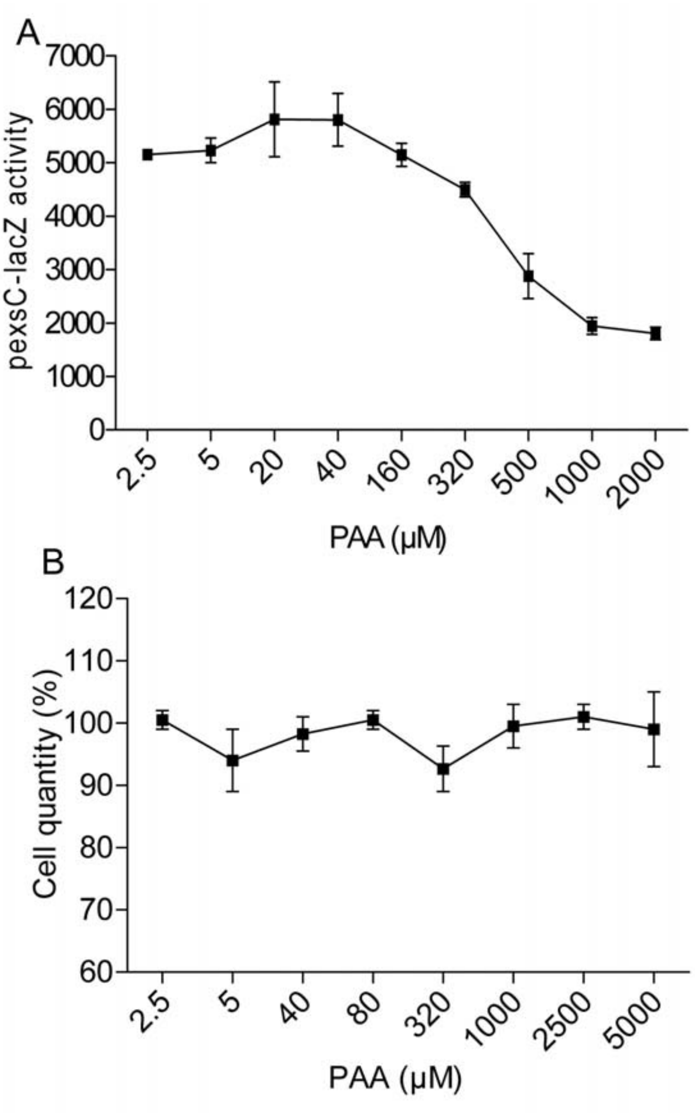
A
7000
6000
5000
4000
3000
2000
1000
0
pexsC-lacZ activity
2.5
5
20
40
160
320
500
1000
2000
PAA (μM)
B
120
110
100
90
80
70
60
Cell quantity (%)
2.5
5
40
80
320
1000
2500
5000
PAA (μM)

Fig S6.
